# Impact of genetic variation on three dimensional structure and function of proteins

**DOI:** 10.1371/journal.pone.0171355

**Published:** 2017-03-15

**Authors:** Roshni Bhattacharya, Peter W. Rose, Stephen K. Burley, Andreas Prlić

**Affiliations:** 1 Bioinformatics and Medical Informatics, San Diego State University, San Diego, California, United States of America; 2 RCSB Protein Data Bank, San Diego Supercomputer Center, University of California San Diego, La Jolla, California, United States of America; 3 RCSB Protein Data Bank, Department of Chemistry and Chemical Biology, Center for Integrative Proteomics Research, Institute for Quantitative Biomedicine, and Cancer Institute of New Jersey, Rutgers, The State University of New Jersey, Piscataway, New Jersey, United States of America; 4 Skaggs School of Pharmacy and Pharmaceutical Sciences, University of California San Diego, La Jolla, California, United States of America; University of Michigan, UNITED STATES

## Abstract

The Protein Data Bank (PDB; http://wwpdb.org) was established in 1971 as the first open access digital data resource in biology with seven protein structures as its initial holdings. The global PDB archive now contains more than 126,000 experimentally determined atomic level three-dimensional (3D) structures of biological macromolecules (proteins, DNA, RNA), all of which are freely accessible *via* the Internet. Knowledge of the 3D structure of the gene product can help in understanding its function and role in disease. Of particular interest in the PDB archive are proteins for which 3D structures of genetic variant proteins have been determined, thus revealing atomic-level structural differences caused by the variation at the DNA level. Herein, we present a systematic and qualitative analysis of such cases. We observe a wide range of structural and functional changes caused by single amino acid differences, including changes in enzyme activity, aggregation propensity, structural stability, binding, and dissociation, some in the context of large assemblies. Structural comparison of wild type and mutated proteins, when both are available, provide insights into atomic-level structural differences caused by the genetic variation.

## 1. Introduction

With the ever-growing importance of genomics for human health, considerable efforts have been devoted to linking human phenotypes to genotypic variations at the nucleotide level and changes in 3D protein structure [1,2]. Genetic variation can cause changes in phenotype if expression levels are altered or pre-mRNA splicing is affected. Sequence changes at the amino acid level influence the shape, function, or binding properties of a given protein. Of particular interest when analyzing genome-sequencing data are Single Nucleotide Variations (SNVs). Most SNVs are neutral or have no effect on human health or embryonic development [3,4]. Certain SNVs, however, may be useful for predicting individual responses to particular drugs, susceptibility to other exogenous factors such as environmental toxins, or risk of developing disease [4,5,6]. Identification of an SNV giving rise to a phenotype is a challenging problem, owing to the complexity of human biology. Association studies are often used to identify the SNV (or SNVs) giving rise to complex phenotypes [7], relying on genetic variations among affected individuals to detect association of the variation with a trait (or phenotype). Such studies generally concentrate on associations between point mutations and phenotypic traits or diseases [8]. However, Genome Wide Association Studies (GWAS) require screening of large numbers of markers [9,10,11], and correlation of a given SNV with a particular phenotype does not *per se* prove causality. Although genome wide studies provide insights into the genetic basis of human disease, they have explained relatively little of the heritability of many complex traits. This shortcoming has raised the question of where the 'missing heritability' of complex diseases might be found [12].

One way to analyze large datasets of genetic variation is to use bioinformatics tools to filter the data [9]. Computational methods such as SIFT [13,14], Polyphen-2 [15], or MAPP [16] classify SNVs according to negative, neutral, or positive effects on the structure or function of the protein. Several algorithms even attempt to estimate the change in the free energy of stabilization of protein structure, due to single sequence changes, e.g., DUET [17], Mupro [18], and I-Mutant2.0 [19]. A method developed by Topham and colleagues, Site Directed Mutator (SDM), utilizes an approach analogous to the thermodynamic cycle [20,21]. Alternative analytic tools use sequence conservation of a particular amino acid within a protein family, or search for a distinct protein structure feature to predict whether a substitution affects function, such as SIFT or Sorts Intolerant From Tolerant substitutions [13,14,21]. Other bioinformatics tools based on evolutionary principles that predict the effect of coding variants on protein function, including PANTHER [22,23], HMMER/LogR.E-value [24], Condel [25], and several others [26,27,28,69]. Custom databases, including SAAP [27], PolyDoms [28], topoSNP [29], SNPeffect [30], SNPs3D [31], MutDB [32], FATHMM [33] and LS-SNP [34], provide links between SNVs and protein sequence/structure data and/or cellular processes such as localization, phosphorylation, and glycosylation. The National Library of Medicine NCBI supports the tranSNP tool, which permits display of the location of a SNV on the genome [35]. ENSEMBL offers the Variant Effect Predictor [36]. The resources described above use one of the six popular training dataset enumerated in Table 1. Notwithstanding the sophistication of these and other approaches, there is always a question as to whether predictions therefrom can be relied on, because there are numerous examples of discordance among single mutation prediction methods.

**Table 1:** Six popular training datasets for tools predicting the effect of single point mutations.

Dataset	Compiled from	Size	Reference
**MutPred**	SwissProt and HGMD	65,657	[37]
**SNPs&GO**	SwissProt	38,460	[38]
**PON-P**	dbSNP, PhenCode, IDbases and 16 individual locus-specific databases	39,670	[39]
**HumVar**	SwissProt and dbSNP	41,918	[40]
**Humsavar**	SwissProt/UniProt	36,994	[41]
**PredictSNP**	SwissProt/UniProt	43,883	[42]

Arguably, the most informative source of data that can explain what is causing a particular phenotype is the availability of a 3D experimentally-determined structure that contains atomic level insight into the consequences of a particular genomic variant. The RCSB Protein Data Bank (RCSB PDB) [35] enables open access to the Protein Data Bank archive of experimental structures of biological macromolecules without limitations on usage. The PDB is one of the most widely used digital data resources in biology and biomedicine worldwide. The RCSB PDB provides deposition, annotation, query, analysis and visualization tools, and educational resources for use with the PDB archive [43]. All of the 3D macromolecular structure data in the PDB were obtained by one of three experimental methods: X-ray Diffraction (~89%), solution Nuclear Magnetic Resonance (NMR) (~10%), or Electron Microscopy (<1%). PDB structures provide atomic level detail with which to analyze the structural effects of non-synonymous coding SNVs.

Knowledge of the 3D structure of a gene product is beneficial in predicting and understanding both function and role in disease. However, most studies that analyze the relationship between point mutations and experimentally observed 3D protein structure published to date have been restricted to individual proteins or single diseases. There is a paucity of quantitative analyses of the consequences of SNVs on 3D protein structure going beyond the realm of prediction [44].

The goal of this study is to improve our understanding of the relationship between point mutations and experimentally observed consequences in 3D. We identified a benchmark dataset of protein structures that contain well-characterized point mutations for which 3D atomic coordinates are available from the PDB. We manually analyzed 374 human protein structures and SNVs. Herein, we present a detailed overview about the observed effects of SNVs on the structure, function, stability, and binding properties of proteins.

## 2. Methodology

### 2.1. Construction of the dataset

The data set used in this paper is a semi-automatically derived and hand-curated collection of proteins, each of which possess an amino acid that has been changed by a SNV and 3D atomic coordinates are available in the PDB.

To assemble this data set,

1. We identified 2596 structures extant in the PDB for which non-synonymous SNV could be mapped *via* LS-SNP/PDB [34]. For each PDB entry, the amino acid sequence of the crystallized protein experimentally observed in 3D differs from the corresponding UniProt sequence at the position of the variation.
2. From these 2596 structures, we selected only those structures for which the dbSNP mutation information matched information coming from UniProt and the 3D structure. For example, rs28933981, the change in dbSNP is T→M and in PDB: 1BZE, the sequence difference in the structure is also T→M, and this case was included in our dataset. In contrast, the dbSNP database entry for SNV rs128620185 reports R→H, but in the PDB archive (1BTK) the experimentally observed sequence difference is R→C. This case was excluded from our dataset, because it does not correspond to the reported R→H SNV.
3. After filtering for database inconsistencies, we removed mappings of the same SNV to multiple PDB entries, ensuring that each SNV is only represented once. When multiple PDB entries with the same mutation are available, preference was been given to structures determined by X-ray crystallography. In a few cases it was not possible to do so, and the dataset contains 49 structures determined by NMR. (see supplemental files S1 and S2 Figs).

This rigorous procedure yielded a final benchmark dataset of 374 unique human SNVs, each corresponding to a different PDB entry for which 3D atomic level coordinates are available. When filtering by protein sequence identity, the dataset contains 334 unique PDB structures, documenting that we accepted some limited redundancy when constructing the dataset. Each of the 374 SNVs are described in independent experiments, and all such cases were retained in the dataset. See supplemental file S1 File for the complete dataset.

### 2.2. Manual annotation of SNVs

To enumerate the consequence(s) of a given SNV on a gene product, we systematically reviewed the available literature to identify experimentally verified functional effects. We also performed searches in several databases (see below). For each SNV, we extracted the following information from literature and from databases:

1. The *position* of the SNV on the 3D protein structure in the PDB (present on the surface vs. buried in the interior), estimated with BioJava surface accessibility calculations [70].
2. Whether the amino acid substitution falls within *Loop* vs. *Alpha_helix* vs. *Beta_strand* secondary structure, determined from secondary structure annotations obtained from PDB [35,43].
3. What *effect* or *consequence* does the SNV have on the protein?

We classified mutations, whether they affect *Activity of a protein* vs. its *Stability* vs. *Binding* vs. *Assembly* vs. *Rearrangement* (local conformational changes). The 374 PDB structures, which reflect the consequences of a particular SNV in this dataset, may contain other point mutations. Such differences may be neutral or the result of intentional mutations to aid in crystallization, etc. The dataset used herein contains only literature described and phenotype causative SNVs that have been linked to structural change(s) at the level of the protein. In many cases, these proteins were deliberately crystallized with a view to understanding the structural consequences of the sequence variation. To determine the frequency with which a SNV occurs in a population, we consulted the NHLBI Exome Sequencing Project (ESP) Exome Variant server [43,44] and dbSNP [34]. SNVs with Minor Allele Frequency (MAF, referring to the frequency at which the least common allele occurs in a given population) at < 1% are considered *Rare*, with the remainder classified as *Common* SNVs.

Databases and servers used in this work were as follows:

- **RCSB PDB**—The RCSB Protein Data Bank [35,43] is the United States regional data center for the Worldwide Protein Data Bank (wwpdb.org), which manages the single global PDB archival repository of experimental 3D structural data of biological macromolecules.
- **LS-SNP/PDB**–Is a web-tool for the annotation of human SNPs. It contains an automated pipeline that systematically maps human non-synonymous SNPs onto PDB structures [34].
- **dbSNP**—The Single Nucleotide Polymorphism Database (dbSNP) [36] is an archive for genetic variation within and across different species developed and hosted by National Center for Biotechnology Information (NCBI) in collaboration with National Human Genome Research Institute (NHGRI). The database contains information about SNPs, short deletion and insertional polymorphisms (indels/DIPs), microsatellite markers and short tandem repeats (STRs), multi nucleotide Polymorphisms (MNPs), heterozygous Sequences, and named variants [36].
- **NHLBI Exome Sequencing Project (ESP) Exome Variant Server—**Contains a large collection of well-phenotyped US populations [45,46].
- **PubMed**—PubMed contains more than 23 million abstracts for biomedical literature from MEDLINE, life science journals, and online books [47].

#### 2.2.1. Software tools for mapping of genetic variation to protein sequence and 3D structure

To enable deeper analysis of genetic variation in the context of protein sequence and 3D structures, we developed tools to facilitate mapping of any genetic location onto corresponding protein sequences and 3D protein structures [43]. These tools are available from the RCSB PDB website [71] and were used to verify the integrity of the benchmark data assembled for this study.

- **Mapping tool from human genomic position to protein sequence and 3D structure**—This tool allows to map coordinates from the human reference assemblies versions 37, or 38 (as provided by the Genome Reference Consortium) to the correct UniProt isoforms and 3D structures. http://www.rcsb.org/pdb/chromosome.do
- **Human Gene View**—This genome browser supports navigation of the human genome and investigating the relationship between PDB archival entries and genes.
- **Protein Feature View**—Provides a rich graphical summary of protein sequence features, including identification of genomic positions mapped to protein sequences.
- **3D Viewer**—The PV (Protein Viewer) enables highlighting of genomic positions mapped to protein structures in 3D.

### 2.3. Categories for assigning effects of SNV

The following categories were used to classify the effects of SNVs at the level of the protein:

1. Activity—The SNV causes increase, decrease, or complete loss of protein activity.
2. Aggregation—The SNV renders the protein aggregation prone.
3. Stability—The SNV causes a change in protein stability. It may make the protein susceptible to proteolytic cleavage, or cause a change in thermal inactivation temperature, or cause a change in the energy of stabilization of the protein. It can also lead to destabilization of a protein oligomer, loss of packing or hydrophobic interactions, or change a mode(s) of protein-protein interaction.
4. Binding/Dissociation–The SNV leads to changes in affinity for a known binding partner, or alterations in association or dissociation kinetics. It can also cause structural changes in the binding site or affect specificity for a binding partner(s).
5. Assembly—The SNV affects the oligomeric assembly properties of the protein.
6. Rearrangement—The SNV causes local structural rearrangements (conformational changes) in the neighborhood of the amino acid change arising from the SNV.

## 3. Results and discussion

### 3.1. Location of SNVs within 3D structures

**Fig 1:**
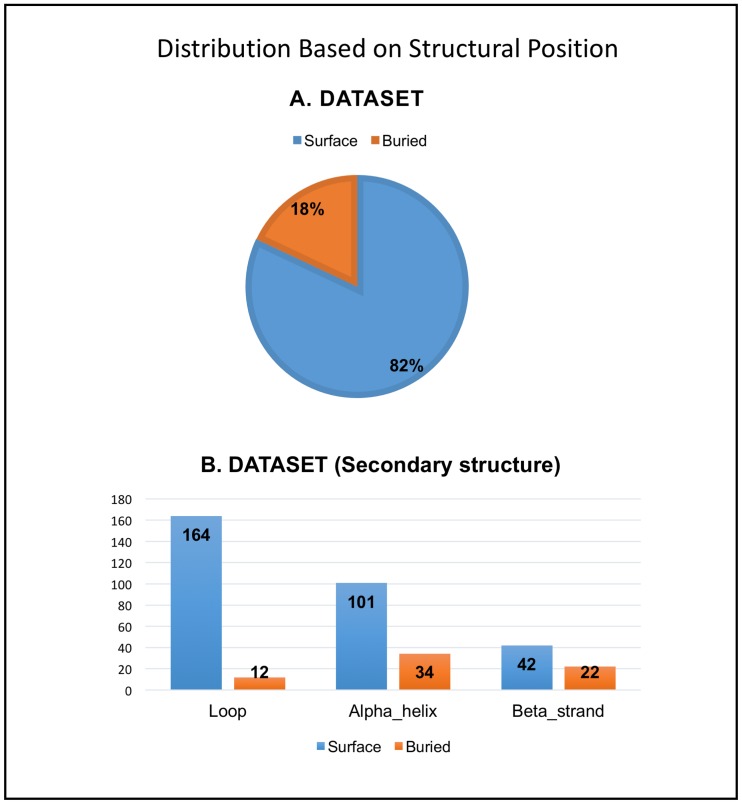
Distribution of SNVs based on structural position. A. Distribution of the SNVs in our benchmark dataset based on structural position (Piechart). There are two broad categories, *Surface* (residue) or *Buried* (residue). B. Distribution of the SNVs in the dataset based on secondary structural location (Bar graph). The two broad categories, *Surface* (residue) or *Buried* (residue) are further categorized into *Loop*, *Alpha_helix* and *Beta_strand* based on the secondary structural element to which the SNV maps.

We first investigated whether it is possible to identify patterns concerning sites at which point mutations occur. Specifically, we determined the position amino acid change caused by the SNV within the 3D structure available from the PDB. Structural locations of the SNVs were then manually categorized into 2 main groups: *Surface* and *Buried*, by analyzing the biological assembly (3D oligomeric structure) of the protein. We observed that 79% of the SNVs (297 of 374) lie on the protein surface and the remaining 21% (77 of 374) were buried in the interior of the protein (Fig 1A). For reference, surface and buried residues comprise 71% and 29%, respectively, for all residues in all of the structures in the dataset.

*Surface* and *Buried* categories were further subcategorized into *Loop*, *Alpha_helix* and *Beta_strand* according to the secondary structural context of each SNV related change within the corresponding PDB structure. Considering the secondary structures, the expected distribution in our dataset is 46% Alpha_helix, 24% Beta_strand and 30% Loop regions.

**Fig 2:**
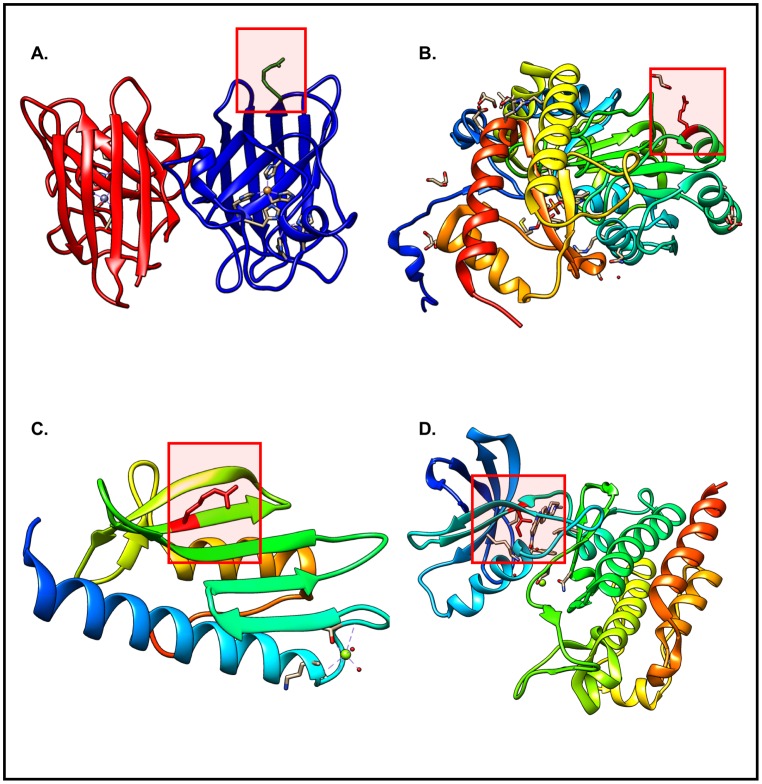
SNV consequences map to various locations within protein structures. A) PDB: 1AZV, SNV: rs121912431 (G37R) is present on the surface of the protein in the highlighted Loop segment, where it causes the neurological disease Lou Gehrig’s disease. B) PDB: 1J04, SNV: rs121908529 (G170R) is present on the surface of the protein in the highlighted Alpha_helix, where it causes hereditary kidney stone disease primary hyperoxaluria type 1. C) PDB: 3S5E, SNV: rs138471431 (W155R) is present on the surface of the protein in the highlighted Beta_sheet, where it causes the neurodegenerative disease Friedreich’s ataxia. D) PDB: 2V7A, SNV rs121913459 (T315I) is present in the ATP-binding domain and causes resistance to the drug imatinib in patients with chronic myelogenous leukemia.

In the *Surface* category, it was observed that 52% (155 out of 297) of the SNVs map to *Loop* regions compared to ~34% for *Alpha_helix* and ~14% for *Beta_strand*. This finding was not unexpected as amino acid changes in *Loop* regions can often be compensated for without affecting the structure and function of the protein, owing to the flexibility of these polypeptide chain segments. In contrast, for the *Buried* category, ~42% of the SNVs map to *Alpha_helix* vs. ~31% in *Beta_strand* vs. ~27% in *Loop* regions (Fig 1B). Thus, the SNVs in the *Surface* category have a higher likelihood of being found in *Loop regions* when compared to the *Buried* category, wherein SNVs related changes are more likely to be found in *Alpha_helix* and *Beta_strand*. Similar distributions based on structural position and secondary structural elements were observed when comparing the SNVs with unknown structural and functional consequence and SNVs with structural and functional consequence information (see S3 Fig). Representative examples are illustrated in Fig 2.

### 3.2. Consequences of SNVs related changes

By systematically reviewing relevant peer-reviewed literature, we determined that a broad range of possible effects could be attributed to a single residue change. To categorize these findings, we classified responses or consequences due to SNVs as follows: *Activity*, *Aggregation*, *Stability*, *Binding*, *Assembly*, and *Rearrangement* (Section 3.3, Table 2). However, the level of detail with which each of the SNVs related changes have been experimentally characterized varies. For example, functional assays have only been performed for a relatively small number of cases. Data pertaining to functional consequences of the mutation are NOT readily available in the literature for 249 of 374 SNVs (~66%), and data regarding the structural consequences of the SNVs related changes are NOT available for 284 of 374 of SNVs (~75%). Nevertheless, the effects that have been described in the literature are often quite dramatic. Table 3 provides examples of what we do know about the 374 SNV cases comprising our dataset.

**Table 2:** Consequence of SNVs on protein structure and function for a dataset of 374 SNVs for which experimentally obtained atomic level data for the variation is available in the Protein Data Bank. Each SNV can be scored for multiple categories.

**Activity**	52
**Aggregation**	28
**Stability**	58
**Binding**	44
**Assembly**	19
**Rearrangement**	25

**Table 3:** Examples for each SNV related effect category.

**Activity**	rs137852646	Glycyl-tRNA synthetase	2PMF	2ZT5	G526R	Loss of activity	Charcot-Marie-Tooth disease	[50]
**Aggregation**	rs121912442	Cu, Zn superoxide dismutase [HSOD	1N19	4FF9	A4V	Destabilization of protein and formation of aggregates.	Lou Gehrig’s disease	[51]
**Stability**	rs74315351	DJ-1	2RK4	1P5F	M26I	Leads to decrease thermal stability and inactivation.	Rare forms of familial Parkinsonism	[52,54]
**Binding**	rs104894227	HRAS	2QUZ	2CE2	K117R	Increases the rate of nucleotide dissociation and results in constitutive activation of HRAS.	Costello Syndrome	[55]
**Assembly**	rs1141718	Manganese superoxide dismutase	1VAR	1MSD	I58T	The packing defects due to the mutation disrupt the dimer-tetramer equilibrium and favor the dimer over tetramer in solution.	Amylotrophic Lateral Sclerosis	[56]
**Rearrangement**	rs61749389	von Willebrand factor	1IJK	1OAK	I546V	The mutation causes a “Gain of Function” effect and produces a phenotype in which regulation is lost	von Willebrand disease	[57]

Examples for each response category are summarized below in Table 3.

**Fig 3:**
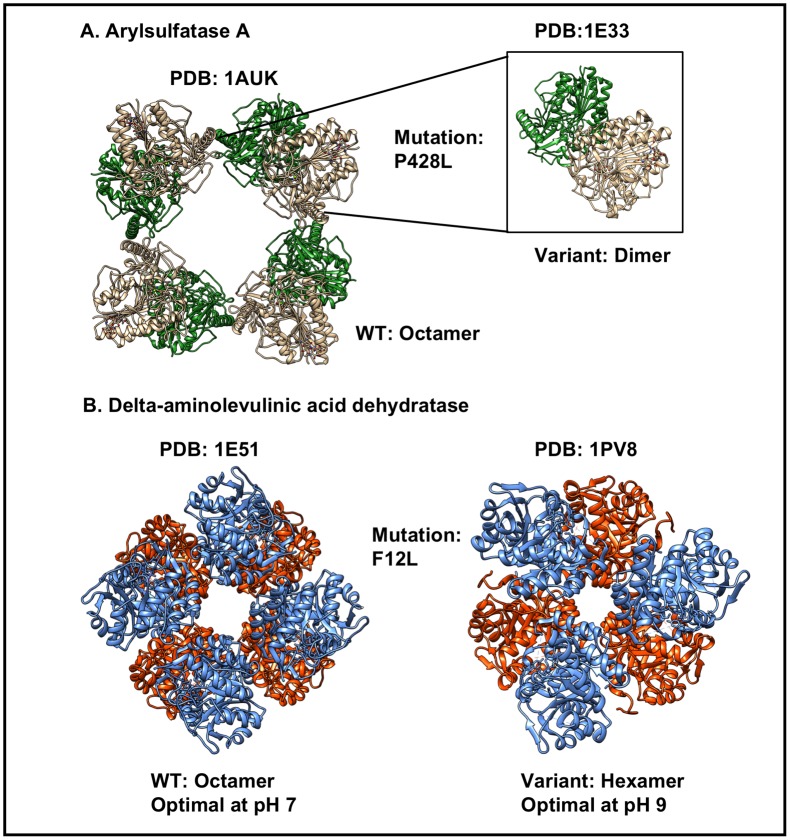
SNVs that affect both protein structure and function. A) The P428L mutant form of Arylsulfatase A adopts an atypical dimeric configuration (instead of the normal octamer), which reduces protein half-life. B) The F12L mutant form of Delta-aminolevulinic acid dehydratase assembles as a hexamer (instead of the normal octamer), which shifts the pH optimum of the enzyme from pH 7→pH 9.

A single residue mutation can have multiple effects on the protein structure and function. Thus, the consequences of a single SNV can affect more than one of the six categories represented in Table 3. Two informative case studies are discussed below:

Arylsulfatase A (gene: ARSA) breaks down sulfatides. The Pro→Leu mutation (P428L) (rs28940893) mapping to amino acid 426 in the PDB structure yields an oligomerization defect (preferred mutant assembly is dimer instead of octamer as for wild-type (Wildtype PDB: 1AUK)) that increases the susceptibility of the protein to degradation by lysosomal cysteine proteinases, leading to severe reduction in half-life [48] and metachromatic leukodystrophy [48]. Therefore, this SNV related change affects both Stability and the protein Assembly (Fig 3A).

Delta-aminolevulinic acid dehydratase (gene: ALAD) catalyzes an early step in tetrapyrrole biosynthesis [49]. The Phe→Leu mutation (F12L) (rs121912984) causes ALAD Porphyria, a rare autosomal recessive disease. Despite of being located far from active site residues 199 and 252 (21.7 and 24.0Å, respectively) this variant changes the preferred protein assembly from octamer to hexamer. In addition, the optimal pH for enzyme activity is shifted from pH 7 (wild-type) to pH 9 in the mutant. The mutant enzyme is barely active under physiological conditions [49]. This SNV was, therefore, categorized as an SNV that affects both enzymatic Activity and the protein Assembly (Wildtype PDB: 1E51) (Fig 3B).

In the following section, we provide a summary of the results for each SNV response category, and discuss several examples in more detail.

#### 3.2.1. Activity

**Fig 4:**
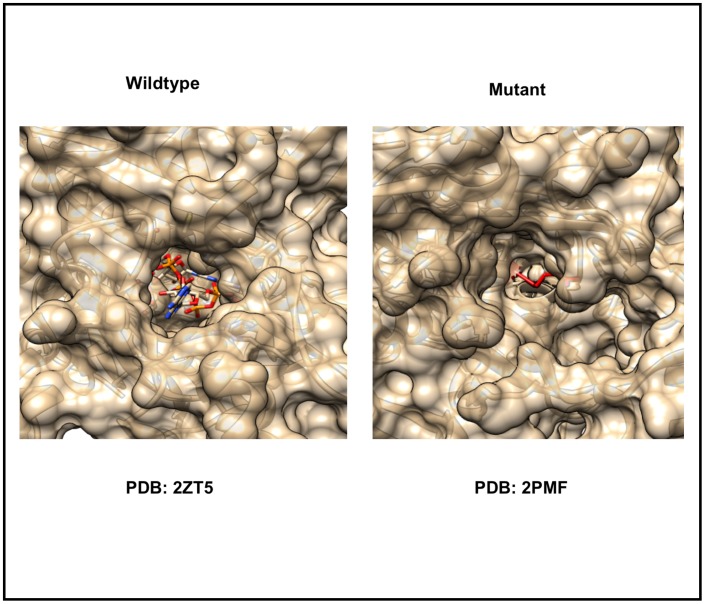
SNV related change that affects enzymatic activity. Semi-transparent, solvent-accessible surface representation of the AMP binding site. For the wild-type structure (PDB: 2ZT5) AMP is bound in the active site (atom type color coded stick figure), while in the mutant structure (PDB: 2PMF) AMP binding is blocked by projection of the arginine sidechain (red) into the active site, thereby blocking substrate ATP binding and inactivating the enzyme.

52 of 374 SNV related changes in our dataset (~14%) either increase or decrease protein activity. In some cases, SNVs lead to complete loss of function. For example, human glycyl-tRNA synthetase (mutant PDB: 2PMF) loses detectable enzymatic activity due to a G526R (rs137852646) mutation, which is causative of Charcot-Marie-Tooth disease [50]. G526 is an evolutionarily conserved residue located in the midst of motif 3 that connects *Beta_strand* β19 with *Alpha_helix* α13. With the exception of the mutation site, the overall structure of the G526R mutant protein is almost identical to that of the wild type (Wildtype PDB: 2ZT5) enzyme (alpha-Carbon atomic position root-mean-square deviation = 0.8Å). Although the G526R change does not disturb the positions of residues comprising the active site, the sidechain of the mutated residue (R526) interdicts access to the active site, thereby inactivating the enzyme [50] (Fig 4).

#### 3.2.2. Aggregation

28 of 374 SNVs related changes in our dataset (~6%) give rise to protein aggregation, which is a hallmark of some neurodegenerative diseases, e.g., Alzheimer's disease (AD), Parkinson's disease (PD), Huntington's disease (HD), amyotrophic lateral sclerosis (ALS), and prion diseases. To exemplify how a single point mutation can induce aggregation, we consider the case of Lou Gehrig’s disease or amyotrophic lateral sclerosis (ALS), which is caused by instability of the Ala→Val (A4V) (rs121912442) mutant of human Cu, Zn superoxide dismutase (HSOD) (mutant PDB: 1N19) [51]. Ala4 is located within a *Beta_strand* adjacent to dimer interface residues and near residues Leu106 and Ile113, which help to stabilize the dimer interface. Leu106 is part of a Greek key super secondary structural motif involved in capping one end of the β barrel. The aliphatic sidechain of Leu106 stabilizes the dimer interface by acting as a cork, which is stabilized by van der Waals interactions with Ala4 and Ile113 [51]. Locations of the sidechains of residues Phe20, Ile113, Leu106, and Ile15 are shifted due to the A4V mutation. This mutation also causes displacement of Leu106 at the one end of the β barrel. Enzymatic activity of the mutant protein is ~50% that of the wild-type (Wildtype PDB: 4FF9). Another consequence of the destabilized A4V mutation is that it facilitates formation of HSOD-containing aggregates, which are believed to be toxic to motor neurons and causative of disease [51].

#### 3.2.3. Stability

**Fig 5:**
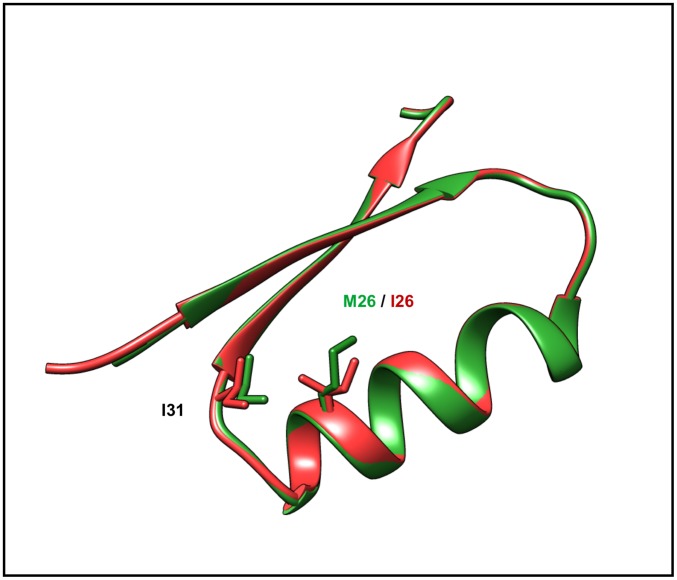
SNV that affects protein structure stability. Disease causing mutation site in protein DJ-1. The wild-type structure (PDB: 1P5F) is depicted in green and the variant (PDB: 2RK4) in red. M26 is a conserved residue in Alpha_helix A located within the hydrophobic core of the protein. The steric clash between I26 and the sidechain of I31 results in a ~0.7 Å displacement of I31 away from I26, resulting in loss of favorable packing contacts involving M26.

58 of 374 SNV related changes in our dataset (~16%) lead to reduced protein stability. A SNV can affect the stability of the protein by making it susceptible to proteolysis or by changing the thermal inactivation temperature. To exemplify how a mutation can influence protein stability, we analyze the following case:

DJ-1 (mutant PDB: 2RK4) is a small conserved protein (189 amino acids), whose absence or inactivation leads to rare forms of familial Parkinsonism in humans [52]. It is also a Ras-dependent oncogene and has been associated with several types of cancers [53]. The Met→Ile (M26I) mutation (rs74315351) decreases thermal stability and enhances formation of DJ-1 aggregates [54]. M26 (Wildtype PDB: 1P5F) is a conserved residue, located in the hydrophobic core of the protein. Although M26 lies near the dimer interface, it does not directly participate in intermolecular protein-protein interactions across the dimer interface. The M26I mutation introduces a β-branched amino acid (isoleucine) into the tightly packed hydrophobic core of the DJ-1 monomer. The steric clash between I26 and the sidechain of I31 displaces the residues slightly and causes loss of optimal packing contacts in the interior of the protein resulting in lower stability [54] (Fig 5).

#### 3.2.4. Binding

44 of 374 SNV related changes in our dataset (~12%) affect ligand or macromolecule binding properties of the protein. A SNV can change the affinity of binding to partners, such as activators, repressors, or substrates. Such changes can also affect the kinetics of interactions with partners or alter binding specificity. Structurally, a SNV can alter the binding site of the protein, which can in turn affect interactions with partner proteins, ligands, etc. The Lys→Arg (K117R) (rs104894227) substitution in HRAS (mutant PDB: 2QUZ) does not alter either intrinsic Ras GTPase activity or responsiveness to GTPase activating proteins, but instead causes constitutive activation of HRAS (and downstream targets) by markedly increasing the rate of GDP dissociation [55]. This mutant HRAS protein activates the RAF/MEK/ERK signaling cascade, leading to growth factor independent cellular proliferation. Although lysine and arginine are both positively charged amino acids, even this conservative substitution results in constitutive activation of HRAS [55]. Clinically, the K117R change in HRAS leads to constant and unchecked cell division causing Costello Syndrome [55], which is a rare genetic disorder affecting many parts of the body.

**Fig 6:**
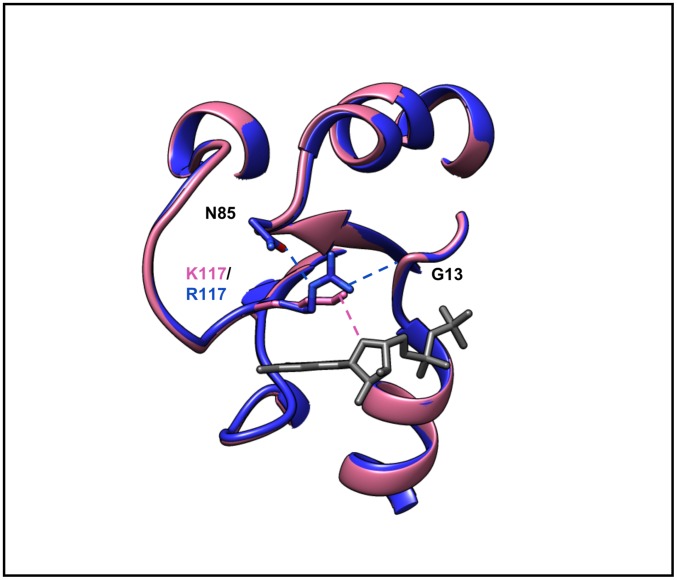
Close-up view of the nucleotide-binding region of Lys117Arg. The mutated residue R117 is stabilized by interactions with the P-loop (Gly13, main-chain CO) and additional interaction with Asn85. Thus the mutated residue causes destabilization of nucleotide binding owing to loss of a direct contact with the ligand. Mutated PDB: 2QUZ(blue) and wild-type PDB: 2CE2 (pink).

The Lys→Arg substitution at position 117 maps to the nucleotide-binding consensus sequence NKXD. In wild-type HRAS (Wildtype PDB: 2CE2), K117 stabilizes nucleotide binding when its aliphatic portion interacting with the base, while its terminal amino group interacts with ribose oxygen O4 of N85 and with a main chain segment (Gly13, CO) from the phosphate binding loop (P-loop)[55]. Destabilization of nucleotide binding is a consequence of subtle rearrangements due to introduction of a larger sidechain capable of making additional polar interactions [55]. (Fig 6)

#### 3.2.5. Assembly

**Fig 7:**
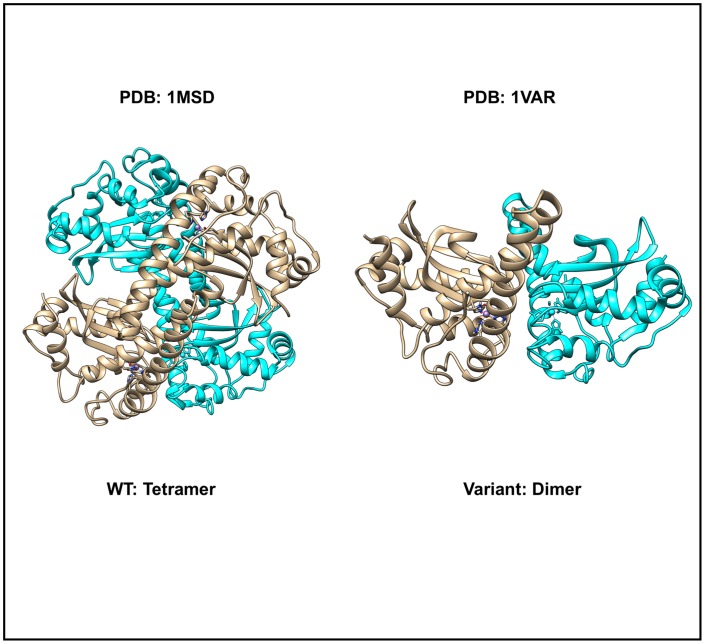
In manganese superoxide dismutase, a SNV can affect protein assembly. The wild type assembly state is tetrameric (left, but due to the mutation mapping to the dimer-dimer interface (in red), the tetrameric structure is not observed in solution (right).

19 of 374 SNVs in our dataset (~5%) change the quaternary structure (oligomeric assembly) of a protein. Mutation of a buried Ile→Thr (I58T) (rs1141718) in the core of the four-helix bundle, which also forms an inter-subunit interface in human manganese superoxide dismutase or MnSOD (mutant PDB: 1VAR), reduces both protein assembly stability and activity. Native human MnSOD is a homotetramer, or more precisely a dimer of dimers. [56]. The I58T mutant form of MnSOD is a dimer, as judged by analytical gel filtration [56]. The native Ile 58 sidechain resides in the dimer-dimer interface, where it helps stabilize the normal tetrameric state of the enzyme (Wildtype PDB: 1MSD). The mutation would introduce a smaller sidechain, Thr58, into the dimer-dimer interface, where a packing defect cavity would be predicted to arise. Hence, disruption of the dimer-dimer interface alters the dimer-tetramer equilibrium, favoring dimer. which may be associated with Amylotrophic Lateral Sclerosis [56] (Fig 7). As predicted from the decrease in thermal stability, the mutant MnSOD is compromised at normal body temperatures. Rapid inactivation of Ile58Thr MnSOD at the elevated temperatures (like during fever and inflammation) would increase superoxide-mediated oxidative damage and perhaps contribute to onset of the diseases.

#### 3.2.6. Rearrangement

**Fig 8:**
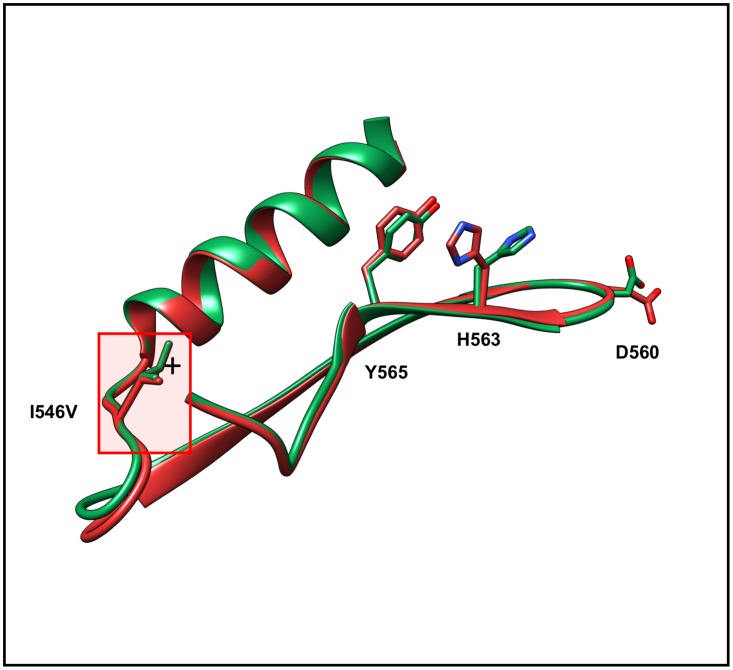
von Willebrand factor (wild-type: green PDBID 1OAK; I546V mutant PDB: 1IJK) with the location of I546V mutation highlighted. Substitution of Ile with Val at position 546 creates a cavity in the hydrophobic core of the I546V mutant structure, which is occupied by a water molecule (denoted by +). The resulting structure perturbation is transmitted through the interior of the protein affecting the locations of the sidechains of Y565, His563, and D560. Collectively, these changes affect Gplb binding, giving rise to von Willebrand’s disease.

25 of 374 SNV related changes in our dataset (~7%) cause significant conformational changes in the vicinity of the mutated residue. The Ile→Val mutation (I546V) (rs61749389) in von Willebrand factor (vWF, mutant PDB: 1IJK) causes the blood clotting disorder von Willebrand disease. The mutation has a “Gain of Function” effect, producing a constitutively active form of vWF that binds platelets in the absence of shear forces [57]. Ile546 lies buried in the hydrophobic core of the protein, close to the A1 domain. (N.B.: vWF binds to the glycoprotein lb or Gplb receptor on platelets *via* interactions with the A1 domain.) In the experimentally determined structure of the mutant protein, a water molecule has insinuated its way into a cavity within the hydrophobic core of the protein, created by the substitution of Ile with the smaller Val sidechain [57]. The presence of the water molecule affects the structure of the A1 domain, which in turn potentiates GpIb binding [57]. The disease-causing mechanism is propagation of conformational changes from the hydrophobic core of the protein to its surface, where Gplb binding is enhanced [57] (Fig 8) (Wildtype PDB: 1OAK).

Such processes have likened to “Rube Goldberg” machines, which were depicted by the Pulitzer Prize winning cartoonist Rube Goldberg. The cartoonist “invented” fictional machines, in which he imagined that a modest perturbation of one part of the machine would lead to big changes at the end of a complicated sequence of concerted interactions (www.rubegoldberg.com/about/).

#### 3.2.7. SNVs not implicated in disease

In the preceding examples, we highlighted 3D protein structural changes arising from SNVs thought to be causative of disease. Many single amino acid variants, however, have effects on macromolecule structure and function that are NOT associated with disease. For example, the T105I variant in Histamine N-Methyltransferase (HNMT) (Mutant PDB: 1JQE) causes a change in temperature dependent specific activity of the protein, but is not known to cause disease [58,59]. In this case, the Ile 105 variant only has significant effects on catalysis at supra-physiologic temperatures (i.e., producing thermal instability at ~50°C), which are incompatible with human life [58]. The identity of the amino acid at position 105 has significant effects on active site structure and dynamics. When visualized in 3D, Ile 105 is seen to make more contacts with other residues in the hydrophobic core than does Thr 105 (Wildtype PDB: 2AOT). Altered packing causes structural rearrangement the polypeptide chain, but does not appear to contribute to disease [59].

Most bioinformatics software tools would predict that the T105I variant is *disease causing* or *not disease causing*, neither of which adequately describe the changes that are actually taking place. Available software tools predict that the T105I variant would have either 1) moderate impact, 2) ~40% chances of being a deleterious mutation, or 3) decreased thermal stability. In fact, the T105I mutation exhibits effects only at supra-physiologic temperatures. There is, therefore, a pressing need for more accurate software prediction tools.

### 3.3. Paucity of structural and functional data for SNVs

For the majority of SNVs represented in our dataset, we found no information about the structural or the functional changes caused by the SNV published in peer-reviewed literature. We grouped all these SNVs into *Unknown_Structural_Consequence* and *Unknown_Functional_Consequence*, respectively. The SNVs that did not have information about the structural consequence (e.g., conformational changes due to the mutation) were grouped in the *Unknown_Structural_Consequence* category. If there is no information in the literature about the functional impact (e.g., affecting the activity or binding) we grouped the SNVs under *Unknown_Functional_Consequence*. For these SNVs no experimental data is available on the effect. Thus, the SNVs whose influence on the structure and function of the protein is not known fall into this category. One possible reason behind the high values in these two categories (249 SNVs in *Unknown_Functional_Consequence* and 284 SNVs in *Unknown_Structural_Consequence*) could be ~70% of missense mutations are thought to be neutral [4]. For reference, 9 of the 374 SNVs well characterized at the protein level have experimental evidence confirming a neutral SNV. We think it likely that most of the 249 or 284 SNVs could also have neutral effect but experimental evidence is required to make any such conclusions.

For a small subset of the 374 PDB entries in our dataset, it was also possible to identify corresponding wild-type structures in the PDB archive. As of late November 2016, 143 PDB entries with SNV related mutations could be matched to a wild type counterpart in the PDB. The supplemental CSV file (S4 File) described in the Data Availability section contains a mapping of PDB IDs for both wild-type and mutant entries, where available.

### 3.4. Special cases

The various categories of SNV consequences enumerated above suffice to describe most observed SNVs. Nevertheless, there are several additional effects that warrant discussion.

**Fig 9:**
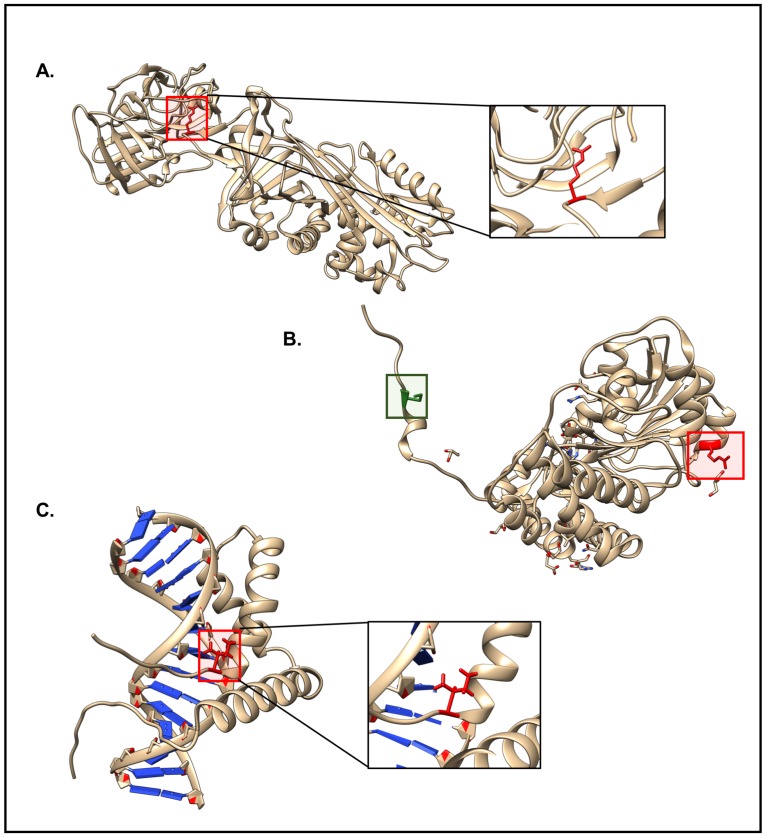
Examples of special cases. A) PDB: 1OPH. The highlighted residue in red represents the mutation (M358R) site. Due to this mutation, alpha1-antitrypsin loses its function as an elastase inhibitor, retains its function as a trypsin inhibitor, and gains a function as a thrombin inhibitor. B) PDB: 1J04. The two highlighted regions represent the two polymorphisms that act synergistically. The highlighted region in green represents P11L polymorphism in AGT whereas the highlighted region in red represents the disease-specific G170R mutation. C) PDB: 1J47. The highlighted red residue represents the M64I in the full-length hSRY sequence, which corresponds to M9I in the given construct and affects the extent of DNA bending.

- **Change of Function** (PDB: 1OPH, SNV ID: rs121912713, Mutation: M358R, Wildtype PDB: 2QUG)—This SNV related change is associated with Alpha1-Antitrypsin Pittsburg, a fatal bleeding disorder [60]. The Met→Arg mutation at position 358 converts alpha1-antitrypsin, an elastase inhibitor, into a thrombin inhibitor. The active site surfaces of elastase and thrombin are sufficiently similar so that wild-type alpha1-antitrypsin Met358 binds to the active site of elastase (which is specific for methionine at the cleavage site) and mutant alpha1-antitrypsin Arg358 binds to the active site of thrombin (which is specific for arginine or lysine at the cleavage site) [60] (Fig 9A).
- **Generation of a mitochondrial targeting sequence** (PDB: 1J04, SNV ID: rs121908529, Mutation: G170R, Wildtype PDB: 1H0C)—This mutation is associated with primary hyperoxaluria type 1 autosomal recessive kidney-stone disease, which is caused by peroxisome-to-mitochondrion mistargeting of the liver specific enzyme alanine glyoxylate aminotransferase (AGT). AGT mistargeting occurs in the context of a common polymorphism (P11L) combined with the disease-specific Gly→Arg mutation at position 170 [61,62]. The polymorphism generates a cryptic mitochondrial targeting sequence [63]. When the G170R mutation is present, AGT no longer forms a stable dimer, and the resulting enzyme monomer is able to cross the mitochondrial membrane (Fig 9B). The disease phenotype is caused by depletion of the enzyme within the peroxisome.
- **Changed DNA binding affinity, DNA bending, sex reversal** (PDB: 1J47, SNV ID: rs104894969, Mutation: M9I, Wildtype PDB: 1J47)–This mutation causes 46X,Y sex reversal. M64I (using the full-length hSRY sequence numbering) acts principally by reducing the amount of protein-induced DNA bending [64]. DNA-binding affinity for the mutant protein is reduced by, at most, a factor of 3 relative to that of wild-type; however, the apparent DNA bend angle induced by M9I protein binding is ~20° less for that measured for the wild-type protein-DNA complex [64]. Even this relatively modest change in bending angle can have significant effects on longer-range interactions among other proteins bound near SRY recognition site (Fig 9C) [64].

### 3.5. Frequency in population

One important question of human genetic studies is how the frequency with which a genetic variation can be found in a population is correlated with the risk for a disease. Genetic contributions to disease have been attributed A) to a large number of small-effect common variants across the entire allele frequency spectrum, B) a large number of large-effect rare variants, or C) some combination of genotypic, environmental, and epigenetic interactions [65,66,67].

With the growing adoption of next-generation sequencing technology, the frequency with which a particular variation can be found in a population is being determined for an increasing number of SNVs. In this context, we examined the known population frequencies of the 374 SNVs in our dataset, and correlated observed frequencies with consequence severity data.

**Fig 10:**
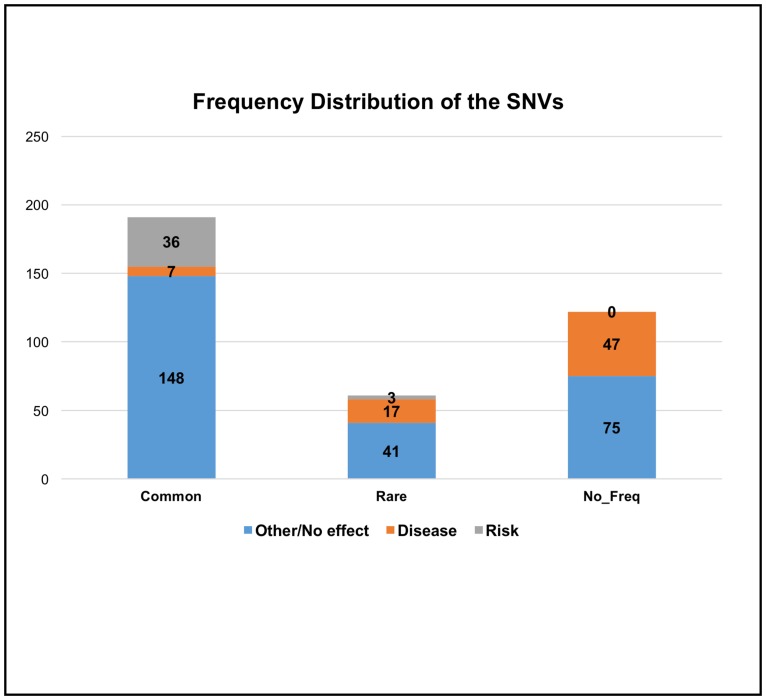
Frequency distribution of the SNVs. Bar graph indicates distribution of SNVs as *Other/No effect* (either neutral or does not cause a disease), *Disease* causing and associated with the *Risk* of developing a disease within each frequency category.

In general, variations are identified as polymorphisms, if they are observed in >1% of the population. If a SNV has a Minor Allele Frequency (MAF) < = 1%, we refer to it as a *Rare* SNV, otherwise as a *Common SNP*. Population frequency data was obtained from the NHLBI Exome Sequencing Project (ESP) Exome Variant Server, which provides data on more than 200,000 individuals in the US, and dbSNP. Among the 374 SNVs we analyzed, 51% (191) were *Common*, 16% (61) were *Rare*, and for 33% (122) no frequency information was available, denoted *No_Freq* (Fig 10). In one case data was discordant between 1000 genomes and ESP. In this case the data was taken from ESP. We further partition this data, based on the severity of the SNV. Where SNVs are associated with a disease, we categorized them as *Disease* causing. SNVs that associated with the risk of developing a disease are grouped under *Risk*. Finally, under *Other/No effect* we identified SNVs that have a neutral effect, or for which no disease related information was available.

*Disease* related SNVs appear to be more frequent in the *Rare* category. In addition, the diseases that have *Common* SNVs generally are much milder and unlikely to be life threatening prior to procreation (such as asthma, or diabetes). Variations in the *No_Freq* category have a large number of disease related SNVs and the frequency distribution is similar to the *Rare* category. We speculate that some of these SNVs are ultra rare SNPs, or the diseases caused by these variations are serious, so a much larger population size might be needed to establish frequencies reliably.

The dataset compiled here contains a mix of large- and small- effect variants. Some of the most striking examples described in this manuscript are rare variations that have large effects on proteins. There are, however, also a large number of SNVs, for which no clear consequence on the 3D protein structure is known. Another possible model to explain these mutations is also the small-effect/common variant hypothesis mentioned above.

## 4. Conclusion

The focus of this study are protein structures in the PDB archive for which 3D structures of genetic variant proteins have been determined. In this context, it is important to note that the contents of the Protein Data Bank do not constitute a representative subset of all proteins. There is selection bias in the PDB in the sense that the availability of the 3D structure of a given protein depends critically on investigator scientific taste, funding trends, technical feasibility, and no small amount of luck at the bench. The data collected here provide important insights into possible structural and functional changes in proteins. But it must be stressed that our work provides a *qualitative* description of possible changes, not a *quantitative* assessment. Notwithstanding the enormous growth in the PDB from 7 to more than 124,000 archival entries, it is simply not possible to provide an accurate account of the consequences of human genetic variation across the human proteome.

Single Nucleotide Variations (SNVs) represent the most common genetic variations observed in humans, accounting for about 90% of sequence differences [68]. In this study, we analyzed the structural and functional effects of single amino acid changes in proteins owing to SNVs. Our analyses of a relatively small dataset of only 374 SNVs underscores the challenges inherent in attempting to understand the consequences of a particular genetic variation at the level of the encoded protein.

Specifically, our results document that the range of possible SNV effects at the protein level are significantly greater than currently assumed by existing software prediction methods, and that correct prediction of consequences remains a significant challenge. In general, most of the software methods that attempt to predict the consequence of SNVs, classify SNVs as either *disease causing* or *not disease causing*. A point mutation may not be causing a disease, but it can still have an effect on the structure and function of the protein. Consequences due to such point mutations often go undetected, as they do not result in a disease phenotype, although they do affect the protein and may perturb normal human physiology.

In addition to the examples described herein, it is easy to imagine that other consequences related to SNV changes will be found as more experimentally determined 3D structures become available and our understanding of protein structure-function relationships continues to grow. For example, the impact of genetic variation on protein-protein interactions is not well represented in the current dataset.

A comprehensive understanding of three-dimensional structure, dynamics, and biophysics of wild-type and mutant proteins will be required to develop better tools that can make accurate predictions regarding the consequences of genetic changes manifested at the atomic level in protein gene products.

## Supporting information

S1 Fig Experimental procedures. Experimental procedures for determining the PDB structures in the dataset of 374 SNVs. 325 SNVs have PDB coordinates determined by X-ray crystallography. 49 have solution NMR structures available in PDB.
(PNG)

S2 Fig Resolution plot. Distribution of the resolution of the X-Ray crystallography PDB structures used for the dataset.
(PNG)

S3 Fig Distribution of SNVs. Distribution of the SNVs in the dataset, for which no structural and functional consequence was found in existing literature, based on structural position and secondary structure elements (left). Distribution based on structural position and secondary structure elements for SNVs with structural and functional consequence information (right).
(PNG)

S1 File Dataset of SNVs. A CSV file containing the annotated dataset that has been used for this manuscript.
(CSV)

## References

[1] Altshuler D, Daly MJ, Lander ES. Genetic Mapping in Human Disease. Science. 2008: 881–8. 10.1126/science.1156409 PMC2694957 18988837

[2] Botstein D, Risch N. Discovering genotypes underlying human phenotypes: past successes for mendelian disease, future approaches for complex disease. Nature Genetics. 2003; 33 Suppl:228–37. 12610532 10.1038/ng1090

[3] Kimura M, Takahata N. Selective constraint in protein polymorphism: study of the effectively neutral mutation model by using an improved pseudosampling method. Proceedings of the National Academy of Sciences of the USA. 1983; 80(4):1048–52. 6573657 10.1073/pnas.80.4.1048 PMC393525

[4] Wang Z, Moult J. SNPs, protein structure, and disease. Human Mutation. 2001; 17(4):263–70. 10.1002/humu.22 11295823

[5] Pirmohamed M, Park BK. Genetic susceptibility to adverse drug reactions. Trends Pharmacological Sciences. 2001; 22(6):298–305. 10.1016/s0165-6147(00)01717-x 11395158

[6] Gamazon ER, Huang RS, Cox NJ, Dolan ME. Chemotherapeutic drug susceptibility associated SNPs are enriched in expression quantitative trait loci. Proceedings of the National Academy of Sciences of the USA. 2010; 107(20):9287–92. 10.1073/pnas.1001827107 20442332 PMC2889115

[7] Risch N, Merikangas K. The future of genetic studies of complex human diseases. Science. 1996; 273(5281):1516–7. 8801636 10.1126/science.273.5281.1516

[8] Hirschhorn JN, Lohmueller K, Byrne E, Hirschhorn K. A comprehensive review of genetic association studies. Genetics in Medicine. 2002: 45–61. 10.1097/00125817-200203000-00002 11882781

[9] Ramensky V, Bork P, Sunyaev S. Human non-synonymous SNPs: server and survey. Nucleic Acids Research. 2002; 30(17):3894–900. 12202775 10.1093/nar/gkf493 PMC137415

[10] Risch NJ. Searching for genetic determinants in the new millennium. Nature. 2000; 405(6788):847–56. 10.1038/35015718 10866211

[11] Lai E, Riley J, Purvis I, Roses A. A 4-Mb high-density single nucleotide polymorphism-based map around human APOE. Genomics. 1998;54(1):31–8. 10.1006/geno.1998.5581 9806827

[12] Eichler EE, Flint J, Gibson G, Kong A, Leal SM, Moore JH, Nadeau JH. Missing heritability and strategies for finding the underlying causes of complex disease. Nature Reviews Genetics. 2010 6;11(6):446–50. 10.1038/nrg2809 20479774 PMC2942068

[13] Ng PC, Henikoff S. Predicting deleterious amino acid substitutions. Genome Research. 2001;11(5):863–74. 10.1101/gr.176601 11337480 PMC311071

[14] Ng PC, Henikoff S. SIFT: Predicting amino acid changes that affect protein function. Nucleic Acids Research. 2003;31(13):3812–4. 12824425 10.1093/nar/gkg509 PMC168916

[15] Adzhubei I, Jordan DM, Sunyaev SR. Predicting functional effect of human missense mutations using PolyPhen-2. Current Protocols in Human Genetics. 2013;(SUPPL.76). 10.1002/0471142905.hg0720s76 PMC4480630 23315928

[16] Stone EA, Sidow A. Physicochemical constraint violation by missense substitutions mediates impairment of protein function and disease severity. Genome Research. 2005;15(7):978–86. 10.1101/gr.3804205 15965030 PMC1172042

[17] Pires DE, Ascher DB, Blundell TL DUET: a server for predicting effects of mutations on protein stability using an integrated computational approach. Nucleic Acids Research. 2014 7;42(Web Server issue):W314–9. 10.1093/nar/gku411 24829462 PMC4086143

[18] Cheng J, Randall A, Baldi P. Prediction of protein stability changes for single-site mutations using support vector machines. Proteins. 2006;62(4):1125–32. 10.1002/prot.20810 16372356

[19] Capriotti E, Fariselli P, Calabrese R, Casadio R. Predicting protein stability changes from sequences using support vector machines. Bioinformatics. 2005;21 Suppl 2:54–8. 10.1093/bioinformatics/bti1109 16204125

[20] Topham CM, Srinivasan N, Blundell TL. Prediction of the stability of protein mutants based on structural environment-dependent amino acid substitution and propensity tables. Protein Engineering. 1997;10(1):7–21. 9051729 10.1093/protein/10.1.7

[21] Burke DF, Worth CL, Priego E-M, Cheng T, Smink LJ, Todd JA, et al Genome bioinformatic analysis of nonsynonymous SNPs. BMC Bioinformatics. 2007;8:301 10.1186/1471-2105-8-301 17708757 PMC1978506

[22] Mi H, Muruganujan A, Thomas PD. PANTHER in 2013: Modeling the evolution of gene function, and other gene attributes, in the context of phylogenetic trees. Nucleic Acids Research. 2013;41(D1). 10.1093/nar/gks1118 PMC3531194 23193289

[23] Thomas PD, Kejariwal A. Coding single-nucleotide polymorphisms associated with complex vs. Mendelian disease: evolutionary evidence for differences in molecular effects.Proceedings of the National Academy of Sciences of the USA. 2004;101(43):15398–403. 10.1073/pnas.0404380101 15492219 PMC523449

[24] Clifford RJ, Edmonson MN, Nguyen C, Buetow KH. Large-scale analysis of non-synonymous coding region single nucleotide polymorphisms. Bioinformatics. 2004;20(7):1006–14. 10.1093/bioinformatics/bth029 14751981

[25] González-Pérez A, López-Bigas N. Improving the assessment of the outcome of nonsynonymous SNVs with a consensus deleteriousness score, Condel. American Journal of Human Genetics. 2011;88(4):440–9. 10.1016/j.ajhg.2011.03.004 21457909 PMC3071923

[26] Choi Y, Sims GE, Murphy S, Miller JR, Chan AP. Predicting the Functional Effect of Amino Acid Substitutions and Indels. PLoS One. 2012;7(10). 10.1371/journal.pone.0046688 PMC3466303 23056405

[27] Al-Numair NS, Martin a C. The SAAP pipeline and database: tools to analyze the impact and predict the pathogenicity of mutations. BMC Genomics [Internet]. BioMed Central Ltd; 2013;14 Suppl 3:S4. 10.1186/1471-2164-14-S3-S4 PMC3665582 23819919

[28] Jegga AG, Gowrisankar S, Chen J, Aronow BJ. PolyDoms: A whole genome database for the identification of non-synonymous coding SNPs with the potential to impact disease. Nucleic Acids Research. 2007;35(SUPPL. 1). 10.1093/nar/gkl826 PMC1669724 17142238

[29] Stitziel NO, Binkowski TA, Tseng YY, Kasif S, Liang J. topoSNP: a topographic database of non-synonymous single nucleotide polymorphisms with and without known disease association. Nucleic Acids Research. 2004;32(Database issue):D520–2. 10.1093/nar/gkh104 14681472 PMC308838

[30] De Baets G, Van Durme J, Reumers J, Maurer-Stroh S, Vanhee P, Dopazo J, et al SNPeffect 4.0: On-line prediction of molecular and structural effects of protein-coding variants. Nucleic Acids Research. 2012;40(D1). 10.1093/nar/gkr996 PMC3245173 22075996

[31] Yue P, Melamud E, Moult J. SNPs3D: candidate gene and SNP selection for association studies. BMC Bioinformatics. 2006;7:166 10.1186/1471-2105-7-166 16551372 PMC1435944

[32] Dantzer J, Moad C, Heiland R, Mooney S. MutDB services: Interactive structural analysis of mutation data. Nucleic Acids Research. 2005;33(SUPPL. 2). 10.1093/nar/gki404 PMC1160165 15980479

[33] Shihab H a., Gough J, Cooper DN, Stenson PD, Barker GL a, Edwards KJ, et al Predicting the Functional, Molecular, and Phenotypic Consequences of Amino Acid Substitutions using Hidden Markov Models. Human Mutation. 2013;34(1):57–65. 10.1002/humu.22225 23033316 PMC3558800

[34] Ryan M, Diekhans M, Lien S, Liu Y, Karchin R. LS-SNP/PDB: Annotated non-synonymous SNPs mapped to Protein Data Bank structures. Bioinformatics. 2009;25(11):1431–2. 10.1093/bioinformatics/btp242 19369493 PMC6276889

[35] Berman HM, Westbrook J, Feng Z, Gilliland G, Bhat TN, Weissig H, et al The Protein Data Bank. Nucleic Acids Research. 2000;28(1):235–42. 10592235 10.1093/nar/28.1.235 PMC102472

[36] Sherry ST, Ward MH, Kholodov M, Baker J, Phan L, Smigielski EM, et al dbSNP: the NCBI database of genetic variation. Nucleic Acids Research. 2001;29(1):308–11. 11125122 10.1093/nar/29.1.308 PMC29783

[37] Li B, Krishnan VG, Mort ME, Xin F, Kamati KK, Cooper DN, et al Automated inference of molecular mechanisms of disease from amino acid substitutions. Bioinformatics. 2009;25(21):2744–50. 10.1093/bioinformatics/btp528 19734154 PMC3140805

[38] Calabrese R, Capriotti E, Fariselli P, Martelli PL, Casadio R. Functional annotations improve the predictive score of human disease-related mutations in proteins. Human Mutation. 2009;30(8):1237–44. 10.1002/humu.21047 19514061

[39] Olatubosun A, Väliaho J, Härkönen J, Thusberg J, Vihinen M. PON-P: Integrated predictor for pathogenicity of missense variants. Human Mutation. 2012;33(8):1166–74. 10.1002/humu.22102 22505138

[40] Capriotti E, Calabrese R, Casadio R. Predicting the insurgence of human genetic diseases associated to single point protein mutations with support vector machines and evolutionary information. Bioinformatics. 2006;22(22):2729–34. 10.1093/bioinformatics/btl423 16895930

[41] Wu CH, Apweiler R, Bairoch A, Natale D a, Barker WC, Boeckmann B, et al The Universal Protein Resource (UniProt): an expanding universe of protein information. Nucleic Acids Research. 2006;34(Database issue):D187–91. 10.1093/nar/gkj161 16381842 PMC1347523

[42] Bendl J, Stourac J, Salanda O, Pavelka A, Wieben ED, Zendulka J, et al PredictSNP: Robust and Accurate Consensus Classifier for Prediction of Disease-Related Mutations. PLoS Computional Biology. 2014;10(1). 10.1371/journal.pcbi.1003440 PMC3894168 24453961

[43] Rose PW, Prlic´ A, Bi C, Bluhm WF, Bourne PE, Burley SK et al The RCSB Protein Data Bank: views of structural biology for basic and applied research and education. Nucleic Acids Research. 2015 1 28; 43(Database issue): D345–D356. 10.1093/nar/gku1214 25428375 PMC4383988

[44] Arodź T, Płonka PM. Effects of point mutations on protein structure are nonexponentially distributed. Proteins, Structure, Function and Bioinformatics. 2012;80(7):1780–90. 10.1002/prot.24073 22434500

[45] Auer PL, Johnsen JM, Johnson AD et al: Imputation of exome sequence variants into population- based samples and blood-cell-trait-associated loci in African Americans: NHLBI GO Exome Sequencing Project. *American Journal of Human Genetics*. 2012; 91: 794–808. 10.1016/j.ajhg.2012.08.031 23103231 PMC3487117

[46] Exome Variant Server, NHLBI GO Exome Sequencing Project (ESP), Seattle, WA (URL: http://evs.gs.washington.edu/EVS/) [May, 2016]

[47] Sayers EW, Barrett T, Benson DA, et al Database resources of the National Center for Biotechnology Information. Nucleic Acids Research. 2010;38:D5–D16. 10.1093/nar/gkp967 19910364 PMC2808881

[48] von Bülow R, Schmidt B, Dierks T, Schwabauer N, Schilling K, Weber E, Usón I, von Figura K. Defective oligomerization of arylsulfatase a as a cause of its instability in lysosomes and metachromatic leukodystrophy. The Journal of Biological Chemistry. 2002 3 15;277(11):9455–61 10.1074/jbc.M111993200 11777924

[49] Breinig S, Kervinen J, Stith L, Wasson AS, Fairman R, Wlodawer A, Zdanov A, Jaffe EK. Control of tetrapyrrole biosynthesis by alternate quaternary forms of porphobilinogen synthase. Nature Structural Biology. 2003 9;10(9):757–63. 10.1038/nsb963 12897770

[50] Xie W, Nangle LA, Zhang W, Schimmel P, Yang X-L. Long-range structural effects of a Charcot-Marie-Tooth disease-causing mutation in human glycyl-tRNA synthetase. Proceedings of the National Academy of Sciences of the USA. 2007;104(24):9976–81. 10.1073/pnas.0703908104 17545306 PMC1891255

[51] Cardoso RMF, Thayer MM, DiDonato M, Lo TP, Bruns CK, Getzoff ED, et al Insights into Lou Gehrig’s disease from the structure and instability of the A4V mutant of human Cu,Zn superoxide dismutase. Journal of Molecular Biology. 2002;324(2):247–56. 12441104 10.1016/s0022-2836(02)01090-2

[52] Bonifati V, Rizzu P, van Baren MJ, Schaap O, Breedveld GJ, Krieger E, et al Mutations in the DJ-1 gene associated with autosomal recessive early-onset parkinsonism. Science. 2003;299(5604):256–9. 10.1126/science.1077209 12446870

[53] Nagakubo D, Taira T, Kitaura H, Ikeda M, Tamai K, Iguchi-Ariga SM, et al DJ-1, a novel oncogene which transforms mouse NIH3T3 cells in cooperation with ras. Biochemical and Biophysical Research Communications. 1997;231(2):509–13. 10.1006/bbrc.1997.6132 9070310

[54] Lakshminarasimhan M, Maldonado MT, Zhou W, Fink AL, Wilson MA. Structural impact of three Parkinsonism-associated missense mutations on human DJ-1. Biochemistry. 2008;47(5):1381–92. 10.1021/bi701189c 18181649 PMC2657723

[55] Denayer E, Parret A, Chmara M, Schubbert S, Vogels A, Devriendt K, et al Mutation analysis in costello syndrome: Functional and structural characterization of the HRAS p.Lys117Arg mutation. Human Mutations. 2008;29(2):232–9. 10.1002/humu.20616 17979197

[56] Borgstahl GEO, Parge HE, Hickey MJ, Johnson MJ, Boissinot M, Hallewell RA, et al Human mitochondrial manganese superoxide dismutase polymorphic variant Ile58Thr reduces activity by destabilizing the tetrameric interface. Biochemistry. 1996;35(14):4287–97 10.1021/bi951892w 8605177

[57] Fukuda K, Doggett TA, Bankston LA, Cruz MA, Diacovo TG, Liddington RC. Structural basis of von Willebrand factor activation by the snake toxin botrocetin. Structure. 2002;10(7):943–50. 12121649 10.1016/s0969-2126(02)00787-6

[58] Horton JR, Sawada K, Nishibori M, Zhang X, Cheng X. Two polymorphic forms of human histamine methyltransferase: Structural, thermal, and kinetic comparisons. Structure. 2001;9(9):837–49. 11566133 10.1016/s0969-2126(01)00643-8 PMC4030376

[59] Rutherford K, Parson WW, Daggett V. The histamine N-methyltransferase T105I polymorphism affects active site structure and dynamics. Biochemistry. 2008;47(3):893–901. 10.1021/bi701737f 18154359 PMC2905460

[60] Owen MC, Brennan SO, Lewis JH, Carrell RW. Mutation of antitrypsin to antithrombin. alpha 1-antitrypsin Pittsburgh (358 Met leads to Arg), a fatal bleeding disorder. The New England journal of medicine. 1983 p. 694–8. 10.1056/NEJM198309223091203 6604220

[61] Purdue PE, Takada Y, Danpure CJ. Identification of mutations associated with peroxisome-to-mitochondrion mistargeting of alanine/glyoxylate aminotransferase in primary hyperoxaluria type 1. Journal of Cellular Biology. 1990;111(6 PART 1):2341–51. 10.1083/jcb.111.6.2341 PMC2116406 1703535

[62] Lumb MJ, Danpure CJ. Functional synergism between the most common polymorphism in human alanine:glyoxylate aminotransferase and four of the most common disease-causing mutations. The Journal of Biological Chemistry. 2000;275(46):36415–22. 10.1074/jbc.M006693200 10960483

[63] Purdue PE, Allsop J, Isaya G, Rosenberg LE, Danpure CJ. Mistargeting of peroxisomal L-alanine:glyoxylate aminotransferase to mitochondria in primary hyperoxaluria patients depends upon activation of a cryptic mitochondrial targeting sequence by a point mutation.Proceedings of the National Academy of Sciences of the USA. 1991;88(23):10900–4. 1961759 10.1073/pnas.88.23.10900 PMC53039

[64] Murphy EC, Zhurkin VB, Louis JM, Cornilescu G, Clore GM. Structural basis for SRY-dependent 46-X,Y sex reversal: modulation of DNA bending by a naturally occurring point mutation. Journal of Molecular Biology. 2001;312(3):481–99. 10.1006/jmbi.2001.4977 11563911

[65] Cirulli ET, Goldstein DB. Uncovering the roles of rare variants in common disease through whole-genome sequencing. Nature Reviews Genetics. 2010 6;11(6):415–25. 10.1038/nrg2779 20479773

[66] Gibson G. Rare and common variants: twenty arguments. Nature Reviews Genetics. 2012 1 18;13(2):135–45. 10.1038/nrg3118 22251874 PMC4408201

[67] Eichler EE, Flint J, Gibson G, Kong A, Leal SM, Moore JH, Nadeau JH. Missing heritability and strategies for finding the underlying causes of complex disease. Nature Reviews Genetics. 2010 6;11(6):446–50. 10.1038/nrg2809 20479774 PMC2942068

[68] Collins FS, Brooks LD, Chakravarti A. A DNA polymorphism discovery resource for research on human genetic variation. Genome Research. 1998;8(12):1229–31. 9872978 10.1101/gr.8.12.1229

[69] Hecht M, Bromberg Y, Rost B. Better prediction of functional effects for sequence variants. BMC Genomics. 2015; 16 Suppl 8:S1. Epub 2015 Jun 18. 10.1186/1471-2164-16-S8-S1 PMC4480835 26110438

[70] Prlic A et al Biojava: an open-source framework for bioinformatics in 2012. Bioinformatics. 2012; 28(20): 2693–2695. 10.1093/bioinformatics/bts494 22877863 PMC3467744

[71] Prlic A et. Al Integrating Genomic Information with Protein Sequence and 3D Atomic Level Structure at the RCSB Protein Data Bank. Bioinformatics. 2016. 10.1093/bioinformatics/btw547 PMC5167066 27551105

