# Supplementary figures and images for: Impact of genetic variation on three dimensional structure and function of proteins
Source: PLoS One. 2017 Mar 15;12(3):e0171355. doi: 10.1371/journal.pone.0171355 (PMC5351996; doi:10.1371/journal.pone.0171355)

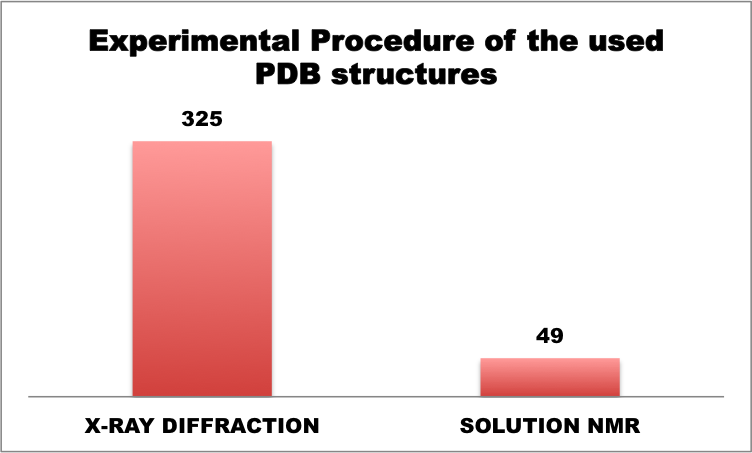

Supplement: S1 Fig — Experimental procedures for determining the PDB structures in the dataset of 374 SNVs. 325 SNVs have PDB coordinates determined by X-ray crystallography. 49 have solution NMR structures available in PDB. (PNG) [file pone.0171355.s001.png]

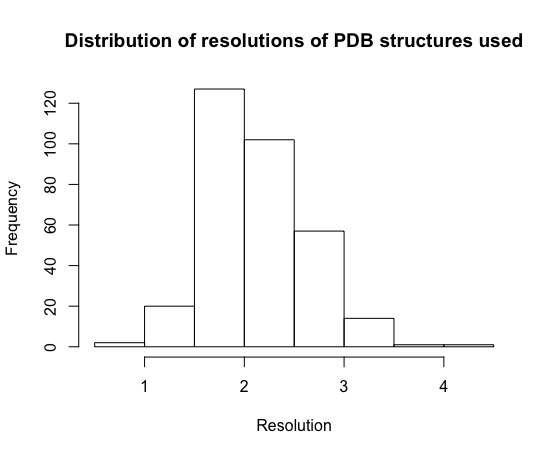

Supplement: S2 Fig — Distribution of the resolution of the X-Ray crystallography PDB structures used for the dataset. (PNG) [file pone.0171355.s002.png]

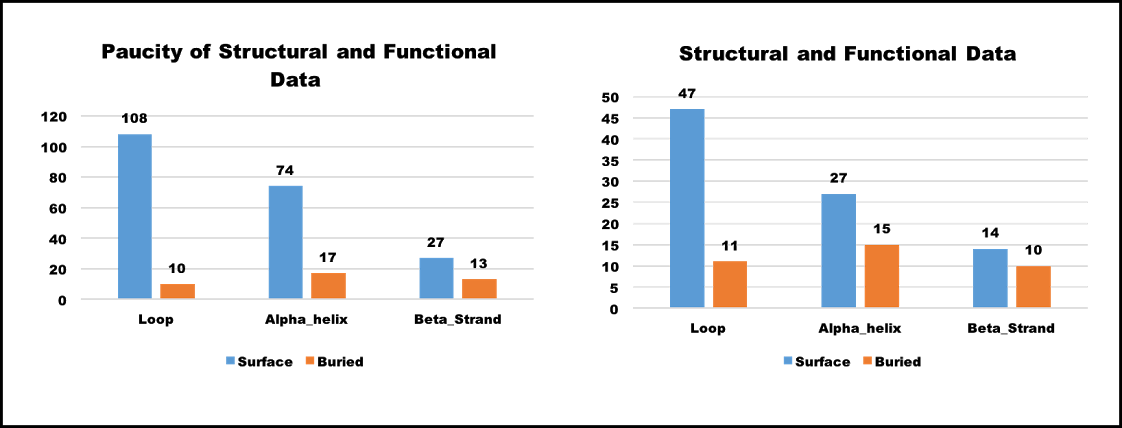

Supplement: S3 Fig — Distribution of the SNVs in the dataset, for which no structural and functional consequence was found in existing literature, based on structural position and secondary structure elements (left). Distribution based on structural position and secondary structure elements for SNVs with structural and functional consequence information (right). (PNG) [file pone.0171355.s003.png]
